# Striving for Perfection: How Stable Is Orthodontic Treatment When Excellent Outcomes Are Achieved? A 9-Year Post-Treatment Retrospective Study

**DOI:** 10.3390/jcm12247692

**Published:** 2023-12-14

**Authors:** Marie A. Cornelis, Arwa Gera, Shadi Gera, Alona Isenshtat, Paolo M. Cattaneo

**Affiliations:** 1 Melbourne Dental School, Faculty of Medicine, Dentistry and Health Sciences, University of Melbourne, 720 Swanston Street, Carlton, VIC 3053, Australia; 2 Private Practice, Yanai St. 4, Jerusalem 9418123, Israel; 3 Private Practice, Irit St. 2, Nahariyya 2221006, Israel

**Keywords:** orthodontic retainers, orthodontics, Little’s irregularity index, PAR index

## Abstract

(1) Aims: The main objective of this retrospective study was to assess the long-term stability of difficult orthodontic treatments treated to an excellent result and to correlate stability to possible prognostic factors. Secondary objectives were to observe the changes in retention protocol over time and to assess Oral Health-related Quality of Life (OHRQoL) after a long-term post-treatment follow-up. (2) Methods: Cases presented for final examination by orthodontic postgraduate students were retrospectively screened for eligibility. Eligible patients were recalled for a post-treatment recall appointment (T2), consisting of a clinical examination and intraoral scan, and were asked to complete the Oral Health Impact Profile-14 (OHIP-14-DK). Gender, age at treatment commencement (T0), treatment modality and duration, and retention protocol were extracted from the records. At T2, the duration of the retention period was recorded, and retainers in place were clinically compared to the original retention protocol. The following variables were assessed on the sets of models at T0, T1 (end of treatment), and T2: arch length and width, overjet and overbite, Dental Aesthetic Index (DAI), Peer Assessment Rating score (PAR), and Little’s Irregularity Index (LII). Multiple regression models were conducted. (3) Results: Eighty-five subjects attended T2. The mean post-treatment follow-up was 9.4 years +/− 2.4. In the upper arch, at T1, 74 patients had a combination of fixed and removable retainers, while at T2, 55 had a fixed retainer only. In the lower arch, at T1, 67 patients had a fixed retainer only, with this number increasing to 76 at T2. From T0 to T1, the PAR score improved by 96.1%, with the improvement remaining at 77.5% at T2. The stability of lower inter-canine and upper inter-premolar widths was significantly correlated with the extent of changes during treatment. The presence of a lower fixed retainer at T2 and a low LII at T1 were prognostic factors for stability. The mean weighted total OHIP-14 score at T2 was very low (1.6 ± 2.4 points). (4) Conclusions: In a sample with an initial high-severity malocclusion and treated to an excellent outcome, long-term stability was very good. Good stability can be retained when a lower fixed retainer is present at T2 and when a low LII is achieved at T1.

## 1. Introduction

The prevalence of malocclusion in adults in the USA is known to exceed 60%, and approximately one child out of two is offered orthodontic treatment in Western countries [1,2]. Over the last few decades, achieving functional and esthetic results has been greatly facilitated by wide improvements in orthodontic materials and techniques. However, once the desired result is obtained, a new challenge starts: to keep this “new” corrected occlusion unchanged. The maintainability of orthodontic treatment achievements on a lifelong basis thus becomes a societal issue. Unfortunately, the biological bases of relapse after orthodontic treatment are still not fully understood [3], retention protocols vary largely from one region to another [4,5,6], and reported failure rates of retainers vary greatly as well [7]. According to the most recent Cochrane Review, there is no ideal retention protocol that can be recommended based on the current evidence available [8].

Long-term stability has been studied for decades [9]. However, most of the numerous studies available on this topic were carried out in the 1990s and early 2000s [5,10,11,12] when the concept of permanent retention was not yet widely accepted and fixed retainers were not routinely used or were removed after a certain number of years. Consequently, long-term post-treatment follow-up in samples with a large proportion of fixed retainers inserted as permanent retention is scarce. Therefore, studying the long-term stability of a population where retainers were not systematically removed after a certain period of time is important.

On the other hand, it has been shown by some authors that long-term stability is correlated to the quality of the orthodontic treatment outcome [13,14]. If the quality of treatment outcome is indeed influencing the long-term stability favorably, it would be of interest to study a sample selected for its high-quality end-of-treatment outcome as it will likely evolve differently in time compared to an average sample. In a context where modern orthodontics is going through a crisis and fixed appliances are slowly replaced by aligners, and because so far it is unclear whether aligners can provide the same level of treatment outcome for difficult malocclusions compared to fixed appliances [15], the question of long-term stability of difficult treatments being treated to an excellent outcome is very pertinent. The latter is even more relevant, given that aligner companies are more frequently targeting general dentists, who might be more prone to offer compromised end-of-treatment results.

For these reasons, the main objective of this retrospective study was to assess the long-term stability of difficult orthodontic treatment treated to an excellent result and to correlate the stability of the outcome to possible prognostic factors related to the patient (age, gender, and initial malocclusion), the orthodontic treatment (treatment duration, extraction or non-extraction) and its outcome (changes in arch length and width, overjet and overbite, Dental Aesthetic Index (DAI) [16], Peer Assessment Rating score (PAR) [17], and Little’s Irregularity Index (LII) [18]), and/or the retention protocol.

The secondary objectives were to observe how the original retention protocols evolved over time and to assess Oral Health-related Quality of Life (OHRQoL) after a long-term post-treatment follow-up.

## 2. Materials and Methods

All cases treated by orthodontic postgraduate students and presented for their final examination at the Section of Orthodontics, Department of Dentistry and Oral Health, Aarhus University, Denmark, from the years 2006 to 2012, were screened for eligibility for this retrospective study. Inclusion criteria were (1) completion of full fixed orthodontic treatment; (2) presence of digital models at pre-treatment (T0) and post-treatment (T1); (3) presence of clinical records (gender, age, treatment modality, duration of treatment, and retention protocol); and (4) presence of 6 anterior teeth in both jaws with normal dental anatomy. Exclusion criteria were patients (1) with cleft lip and/or palate or any other craniofacial syndrome; (2) who had orthognathic surgical correction or maxillary protraction using miniplates; (3) with a history of periodontal disease; (4) with prosthodontic rehabilitation in the anterior region at any time point during the treatment/retention period; or (5) who underwent orthodontic re-treatment after completion of their full fixed appliance treatment. The present retrospective study was given authorization following a written request to the local ethics committee in Denmark (Health Research Ethics Committee-Central Jutland, Denmark, case no. 1-10-72-17-18).

Eligible patients were recalled for a post-treatment recall appointment through an invitation letter sent to their official digital mailbox (eBoks.dk). The following variables were recorded from the patient’s clinical history: gender, age at treatment start, treatment modality (extraction or non-extraction treatment approach), treatment duration, and retention protocol. At the post-treatment recall appointment (T2), the patients had a clinical examination and intraoral scans taken (Trios 3, 3Shape, Copenhagen, Denmark) and were invited to bring their removable retainer(s) if still used. Retainers were clinically checked and subsequently compared to their original retention protocol (T1). In addition, all the patients were asked to complete the Danish version of the Oral Health Impact Profile-14 (OHIP-14-DK) [19]. The time elapsed from end-of-treatment to T2 (named “duration of retention follow-up” hereafter) was recorded.

The digital models at T0, T1, and T2 were imported into the Ortho Analyzer software (3Shape, Copenhagen, Denmark). The following variables were assessed on the sets of models at all of the timepoints by one operator (AG): arch length and width (inter-canine, inter-premolar, and inter-molar distances), overjet and overbite, DAI [16], PAR score [17], and LII [18]. LII was graded into five categories [18]: 0–0.9 mm = “perfect”, 1.0–3.9 mm = “minimal”, 4.0–6.9 mm = “moderate”, 7.0–9.9 mm =“severe”, and ≥10.0 mm =“very severe”. The improvement in PAR score (%) between T0-T1 and T0-T2 was also calculated and categorized according to Richmond et al. [17]: “greatly improved”, “improved”, and “worse or no difference”. The DAI was graded into “no/slight treatment needed”, “treatment elective”, “treatment highly desirable”, and “treatment mandatory” [16].

The weighted total OHIP-14-DK score was calculated, and negative/positive impacts were counted [20]. The OHIP-14 consists of 7 domains (14 questions) scored on a Likert scale ranging from 0 to 4 (0 = ”never”, 1 = ”hardly ever”, 2 = ”occasionally”, 3 = ”fairly often”, and 4 = ”often”), where the lower the score, the better the OHRQoL. The score for each question was then multiplied by its weight and summed into sub-scale scores, which were then again summed up to give the weighted total OHIP-14 score (weighted-standardized method). Responses with “often” or “fairly often” were counted as negative impacts.

The error of the method for all the digital model measurements used in the present study was previously assessed on a separate sample of 26 randomly selected models by two assessors (AG and SG), twice, at a 2-week interval [21].

## 3. Statistical Analysis

Data collection was performed with the Research Electronic Data Capture tools hosted at Aarhus University (REDCap, version 9.4) [22,23]. Statistical analyses were conducted using Stata software (16.1, StataCorp, College Station, TX, USA). Gender, age, duration of treatment, duration of retention follow-up, and treatment characteristics were summarized using descriptive statistics. Multiple regression models were conducted to model the relationship of all outcomes with several prognostics simultaneously. For most of the outcomes, normal distribution support was used to conduct linear regression [24]. With ordered categorical outcomes, a multinomial support distribution was used to conduct ordinal regression [25]. To eliminate the correlation between repeated measures obtained over time on the same subject, a summary statistics approach to repeated measures was adopted by computing derived variables such as “change from T1 to T2”, representing stability. The Bonferroni correction was applied when multiple outcomes were modeled. Predicted means were calculated from the marginal linear mixed models, with derived *p*-values and confidence intervals.

## 4. Results

Three hundred and sixty-four consecutive cases presented for final examination by orthodontic postgraduate students between the years 2006 and 2012 were screened for eligibility. One hundred and forty-nine cases were excluded after applying the eligibility criteria. The eligible 215 cases were contacted for the T2 post-treatment recall appointment. Eighty-five subjects attended the T2 appointment between September 2018 and March 2019. The mean pre-treatment age was 18.1 years, the mean treatment duration was 2.0 years, and the mean post-treatment follow-up was 9.4 years (Table 1). Fourteen patients had been treated with premolar extractions, and seventy-one patients had been treated without extractions (Table 1).

The retention protocol (e.g., fixed and/or removable retainers and the types of removable retainers) at T1 is presented in Table 2. All fixed retainers consisted of round six-stranded stainless steel wires, mostly extending from lateral incisor to lateral incisor in the upper arch and from first premolar to first premolar in the lower arch (Table 3). The changes in retention protocol from T1 to T2 are reported in Table 4. In the upper arch, at T1, most patients (n = 74) had a combination of fixed and removable retainers, while at T2, most had a fixed retainer only (n = 55) (Table 4). In the lower arch, at T1, most patients had a fixed retainer only (n = 67), while this number increased at T2 (n = 76) as most patients who had a combination of fixed and removable retainers at T1 stopped wearing their removable retainer sometime between T1 and T2. With regard to the type of fixed retainers, at T2, almost half of the 13–23 and 34–44 retainers were shortened (Table 3).

**Figure 1:**
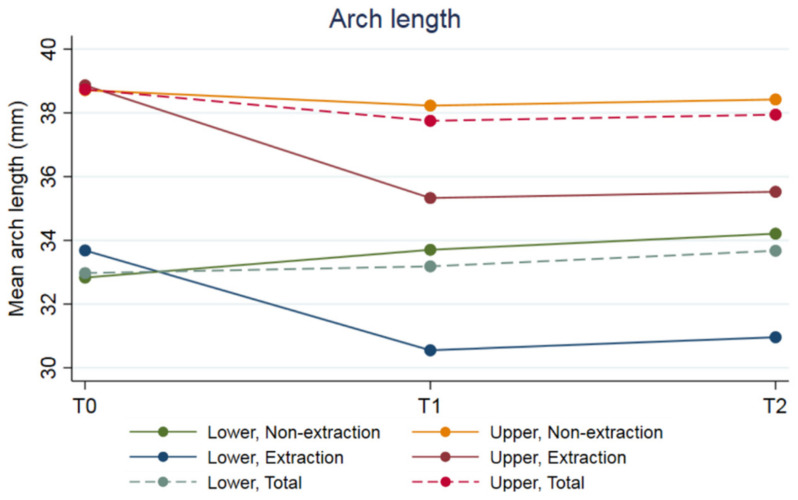

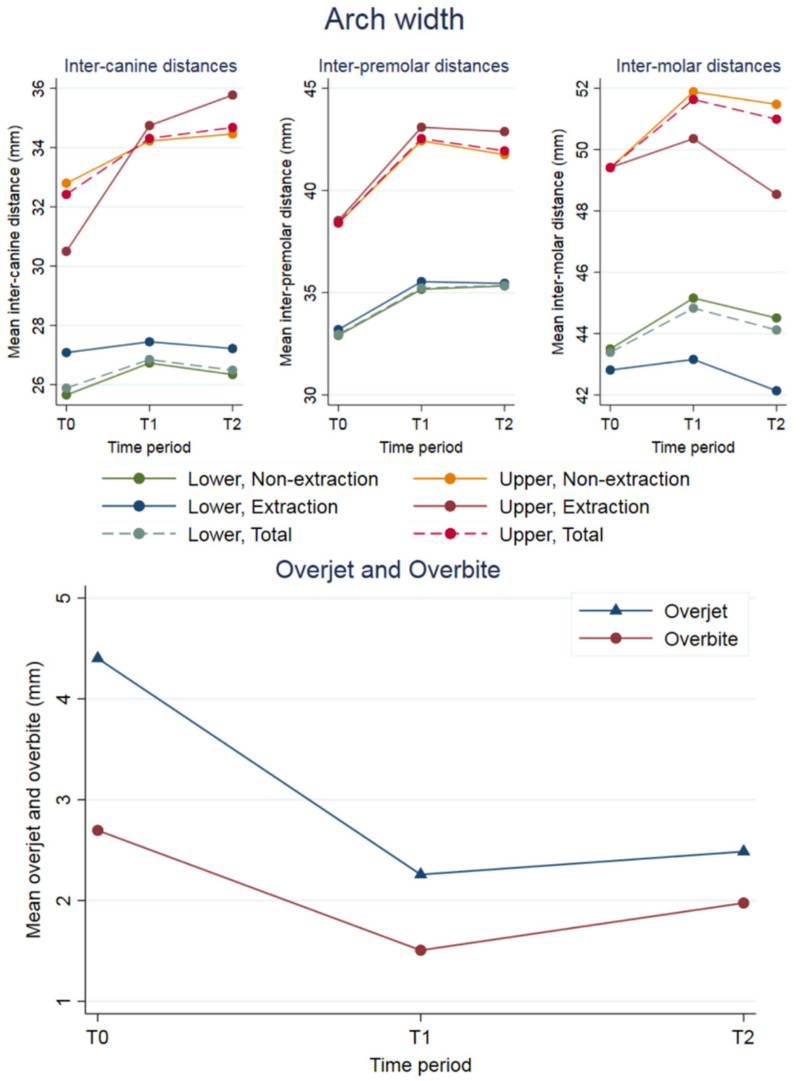
Arch length (**top**) and widths (inter-canine, inter-premolar, and inter-molar distances; (**middle**)), and overjet and overbite (**bottom**).

**Figure 2:**
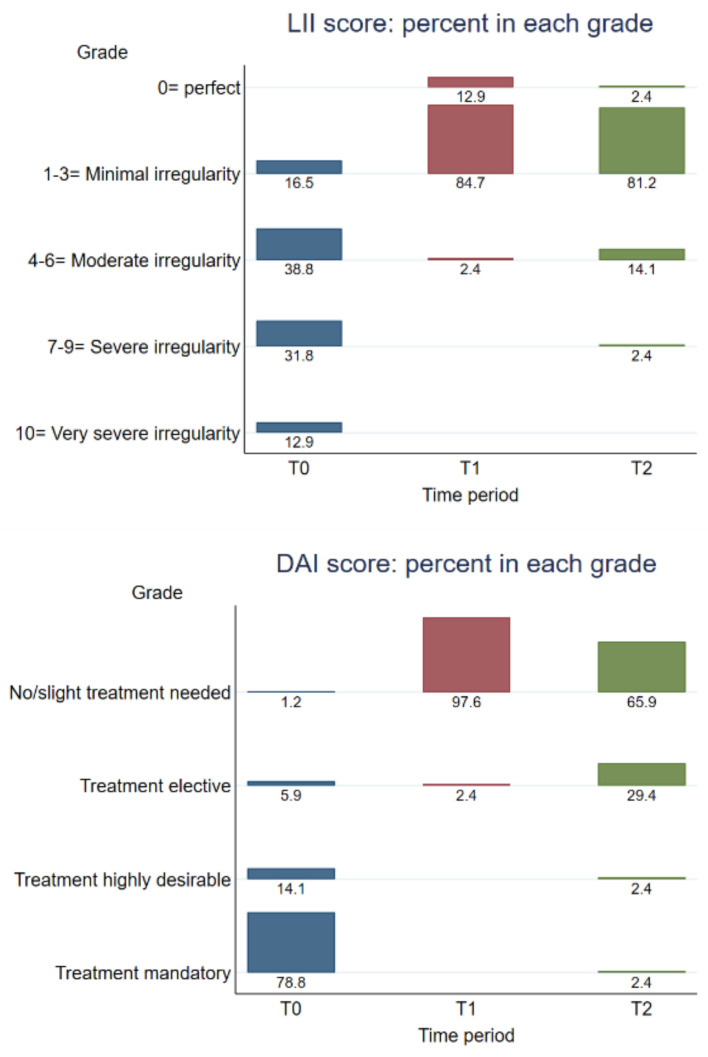
Distribution of LII (**top**) and DAI (**bottom**) scores at T0, T1, and T2.

The arch lengths and widths at T0, T1, and T2 (inter-canine, inter-premolar, and inter-molar distances), overjet, and overbite are shown in Figure 1. The PAR score improved by 96.1% from T0 to T1, with the improvement from T0 to T2 remaining at 77.5% (Table 5). The categories of improvement at T1 and T2 are reported in Table S1. The DAI and LII categories are reported in Figure 2.

### 4.1. Prognostic Factors

The stability (reported as changes occurring from T1 to T2; Table S2) of lower inter-canine and upper inter-premolar widths was significantly correlated with the corresponding changes achieved during treatment (T0–T1): the larger the movements during treatment, the larger the resulting relapse. The stability of overjet, overbite, upper inter-canine, lower inter-premolar, and upper and lower inter-molar widths and arch lengths was not correlated with any prognostic factor. No prognostic factors could be identified for the changes in DAI from T1 to T2. Prognostic factors for changes in PAR scores from T1 to T2 are reported in Table S3. The presence of a lower fixed retainer at T2, the prescription of an upper Essix retainer at T1, and extractions were prognostic factors for changes in PAR scores from T1 to T2. In detail, the presence of a lower fixed retainer at T2 correlated with an 11.1-unit decrease in the difference in PAR scores from T1 to T2 compared to no lower fixed retainer at T2. Compared to an upper Begg retainer, an Essix retainer correlated with a 5.4-unit decrease in the difference in PAR scores from T1 to T2. Compared to extractions, a non-extraction protocol correlated with a 6.0-unit increase in the difference in PAR scores from T1 to T2. The prognostic factors for the LII grade at T2 (Table S3) were the LII at T1 and the presence of a fixed retainer at T2. In detail, an increment of 1 mm in LII index at T1 reduced the odds of being in a better LII grade at T2 by 0.08 (*p* = 0.001), while the presence of a lower fixed retainer at T2 increased the odds of being in a better LII grade at T2 by 733 (*p* = 0.003). All other factors investigated did not appear to be significant prognostic factors (i.e., age, gender, PAR, LII, overjet, and overbite at T0 and T1, treatment duration, duration of retention follow-up, and presence of fixed retainer at T2).

### 4.2. OHRQoL

All subjects answered the 14 questions of the OHIP-14. The mean weighted total OHIP-14 score was very low (1.6 ± 2.4 points). Out of the 1190 responses, the most frequent answer was “never” (85%) (Table 6). The physical pain (D2) and psychological discomfort (D3) responses displayed the highest means (0.5 and 0.4, respectively). There were 17 negative impacts of OHIP-14 in total (3 “often” and 14 “fairly often”). The multiple regression models showed no relationship between the total weighted OHIP-14 score and age, treatment duration, duration of retention follow-up, gender, as well as PAR, DAI, LII, overbite, and overjet at any time point.

## 5. Discussion

Several authors have studied long-term post-treatment stability with similar follow-up periods [5,11,26,27,28,29]. However, to the best of the authors’ knowledge, no study has reported the long-term stability of a sample presenting an initial PAR score as high as in the present study (initial PAR = 32.2), subsequently treated to reach a PAR score as low as in the present study (final PAR = 1.1). Some studies looked at subjects with relatively high initial PAR scores (i.e., PAR = 31.7 [28]; PAR = 29.8 [29]), which are still slightly lower than in the present study and treated to reach a final PAR score higher (PAR = 4.2 [28] and PAR = 6.3 [29]) than that achieved in the present study. Others reported a final PAR score only slightly higher than in the present study (PAR = 2.7 [30]), yet the initial malocclusion was characterized by a lower initial PAR score (PAR = 25.1 [30]). Maia and co-authors reported a low final PAR score (PAR = 1.0), which is very similar to this study; however, the pre-treatment PAR score was considerably lower than in the present study (PAR = 17.0). Hence, the PAR improvement was considerably smaller compared to the present study [31].

Interestingly, the long-term PAR scores of the present study are considerably better than in most long-term studies, suggesting that high-quality post-treatment results could give better chances of preserved outcomes in the long term. This is consistent with the 2015 and 2018 publications by Bjerring and co-authors showing particularly good long-term PAR scores (PAR = 4.4 after 5 years [32]; PAR = 5.1 after 10 years [27]) following very good post-treatment outcomes (PAR = 2.9 [32] and PAR = 2.6 [27]). In the present study, at T2, 82 out of 85 cases were still “greatly improved” or “improved”, with a high percent of improvement still present at T2 (77.5%), which is, again, similar to Bjering and co-authors (79% after 10 years) [27]. In terms of LII, 83.6% of the cases were still “perfect” or presenting “minimal irregularity” at T2 in the present study. The hypothesis that high-quality post-treatment results could provide better chances of maintaining the treatment outcome in the long term is also supported by the fact that the LII at T1 stood out as a statistically significant prognostic factor for long-term stability (*p*-value: 0.001), even though the clinical significance (odds ratio: 0.08) might seem limited.

On the other hand, the maintenance of a high-quality long-term outcome in the present study could also be related to the high numbers of fixed retainers still present at T2 in this sample. The presence of a lower fixed retainer at T2 indeed stood out as a strong positive prognostic factor for stability (PAR score changes from T1 to T2). Similarly, even if short-term prospective studies have failed to show the superiority of fixed retainers over removable retainers, long-term prospective research has indicated that lower fixed retainers offer better long stability compared to removable retainers [33,34], probably because patients tend to wear their removable retainers in the short term, but not indefinitely in the long term.

If fixed retainers warrant long-term stability, orthodontists need failure-free fixed retainers. However, it is known that fixed retainers are associated with high debonding rates [7,35]. Changes in types of fixed retainers, for example, from 13–23 to 12–22 and from 34–44 to 33–43, as reported in the present study, also suggest that the retainers were shortened probably because of breakages or debonding. Besides the risk of failure, multistranded stainless steel fixed retainers, which have been considered the gold standard for decades [36], have been shown to produce unexpected post-treatment changes [37,38] in 1 to 5% of patients. Therefore, if fixed retainers are to be recommended [39], the profession needs to find alternatives to multistranded wires, which, for example, could be offered by new CAD/CAM technology [40].

Another finding that resulted from this study is the fact that in terms of prognostic factors for stability, the changes in lower inter-canine and upper inter-premolar widths from T1 to T2 were significantly correlated with corresponding changes during treatment, which is a confirmation of known facts [9]. The present study also showed that patients initially retained with upper Essix retainers presented less relapse in the long term compared to patients with Begg retainers, but this remains difficult to interpret clinically as the compliance of patients with their removable retainers was not recorded, and most patients had probably stopped wearing their removable retainers several years before T2. Still, it has been shown that vacuum-formed retainers are better accepted by patients compared to Hawley retainers [41], which could explain this finding. Compared to non-extractions, an extraction protocol also correlated with a lesser change in PAR scores from T1 to T2 in the present study, which is in contrast with the literature [9]; however, the low number of extraction cases (14 out of 85) might limit the significance of this finding.

Overall, the OHIP-14 questionnaires in the present study reflected that the patients were still satisfied with the long-term results of their orthodontic treatment 9 years post-treatment. However, the fact that the OHIP-14 questionnaires were only administered at T2, due to the retrospective nature of the present study, is clearly a limitation. Other limitations are possible selection bias due to the sample targeted: (1) the present results are valid for a selected sample of high-quality outcome cases; and (2) attrition (i.e., of the 215 patients recalled, only 85 attended the T2 appointment), which could introduce other selection bias.

## 6. Conclusions

In a sample with an initial high-severity malocclusion treated to an excellent outcome, long-term stability was very good. Good stability can be retained when a lower fixed retainer is maintained in the long term and when excellent results are achieved at T1.

Future long-term prospective studies might overcome the limitations of the present retrospective study.

## Figures and Tables

**Table 1:** Sample characteristics.

	Mean	SD	95% CI
**Age at T0** (years)	18.1	11.8	15.6–20.6
**Duration of treatment** (T0–T1) (years)	2.0	0.4	1.9–2.0
**Post-treatment follow-up** (T1–T2) (years)	9.4	2.4	8.9–9.9
	**Female**	**Male**	
**Gender**	54	31	
**Extraction treatment**	14		
4 premolars	6		
3 premolars	1		
2 premolars	2		
1 premolar	5		
**Non-extraction treatment**	71		

**Table 2:** Types of retainers at T1.

Upper arch	Total fixed retainers	79
	Removable retainers	78
	Begg	18
	Essix	39
	Jensen	6
	Positioner	15
Lower arch	Total fixed retainers	85
	Removable retainers	18
	Essix	6
	Positioner	12

**Table 3:** Changes in type of fixed retainer from T1 to T2 (n).

		**T1**	**T2**
**Upper fixed retainers**	11–21	1	0
	12–22	48	39
	13–23	30	16
	Other	0	10
	total	79	65
**Lower fixed retainers**	43–33	23	29
	44–34	61	36
	Other	1	15
	total	85	80

**Table 4:** Changes in retention protocols from T1 to T2 (n).

		**Upper arch at T2**				
		Fixed Only	Removable Only	Combination (Fixed and Removable)	No Retainers	Total
**Upper arch at T1**	Fixed only	4	0	0	1	5
	Removable only	0	0	0	4	4
	Combination (fixed and removable)	51	3	10	10	74
	No retainers	0	0	0	2	2
	Total	55	3	10	17	85
		**Lower arch at T2**				
		Fixed only	Removable only	Combination (fixed and removable)	No retainers	Total
**Lower arch at T1**	Fixed only	62	1	1	3	67
	Combination (fixed and removable)	14	0	3	1	18
	Total	76	1	4	4	85

**Table 5:** PAR scores at T0, T1, and T2.

	PAR (Points)		
	Mean	SD	95% CI
T0	32.2	9.6	30.1–34.2
T1	1.1	1.7	0.7–1.5
T2	6.6	5.5	5.5–7.8

**Table 6:** Results for the OHIP-14 questionnaire. For the complete list of questions, please see Slade et al. 1997 [20].

		Never	Hardly Ever	Occasionally	Fairly Often	Very Often	Total
Functional limitation	1. pronounce	76	8	1	0	0	85
	2. taste	84	0	0	1	0	85
Physical pain	3. aching pain	61	16	6	1	1	85
	4. uncomfortable	55	19	8	3	0	85
Psychological discomfort	5. self-conscious	75	3	4	3	0	85
	6. tense	55	15	14	1	0	85
Physical disability	7. diet	82	1	1	1	0	85
	8. meals	72	10	2	1	0	85
Psychological disability	9. relax	67	12	4	1	1	85
	10. embarrassed	78	3	3	0	1	85
Social disability	11. irritable	78	7	0	0	0	85
	12. jobs	79	5	0	1	0	85
Handicap	13. life	73	10	1	1	0	85
	14. function	82	2	1	0	0	85
		1017	111	45	14	3	1190

## Data Availability

The data presented in this study are available on request from the corresponding author. The data are not publicly available due to privacy restrictions.

## Supplementary Materials

The following supporting information can be downloaded at https://www.mdpi.com/article/10.3390/jcm12247692/s1, Table S1. Cross-table of PAR improvement categories at T1 and T2 compared to T0; Table S2. Prognostic factors for T1-T2 changes in overjet, overbite, arch width and length; Table S3. Prognostic factors for changes in PAR (T2-T1) and for LII grade at T2.
