# Supplementary material for: Striving for Perfection: How Stable Is Orthodontic Treatment When Excellent Outcomes Are Achieved? A 9-Year Post-Treatment Retrospective Study
Source: J Clin Med. 2023 Dec 14;12(24):7692. doi: 10.3390/jcm12247692 (PMC10743821; doi:10.3390/jcm12247692)
Supplement: Supplementary file 1 [file jcm-12-07692-s001.zip › jcm-2733427-supplementary.pdf]

Table S1. Cross-table of PAR improvement categories at T1 and T2 compared to T0.

		T0-T2			
		**Greatly improved**	**Improved**	**Worse or no difference**	**Total**
**T0-T1**	**Greatly improved**	51	20	1	72
	**Improved**	0	11	2	13
	**Worse or no difference**	0	0	0	0
	**Total**	51	31	3	85

Table S2. Prognostic factors for T1-T2 changes in overjet, overbite, arch width and length.

	Prognostic factor	Coef.	Std. Err.	t	P>|t|	[95% Conf. Interval]	
**LICD T1-T2**	LICD T0	0.41	0.13	3.14	0.003	0.15	0.67
	LICD T0-T1	0.50	0.11	4.38	0.000	0.27	0.72
	- A 1-mm increment in LICD at T0 correlates with a 0.41 mm increase in difference T1-T2.<br>- A 1-mm increment in LICD from T0 to T1 correlates with a 0.49 mm increase in difference T1-T2.						
**UIPD T1-T2**	UIPD T0-T1	0.36	0.12	2.96	0.004	0.12	0.60
	Treatment duration	-1.61	0.51	-3.15	0.002	-2.64	-0.59
	Male	-1.21	0.40	-3.02	0.004	-2.02	-0.41
	- A 1-mm increment in UIPD from T0 to T1 correlates with a 0.36 mm increase in difference T1-T2.<br>- A 1-unit increment in treatment duration correlates with a 1.61 mm decrease in difference T1-T2.<br>- Being male correlates with a 1.2 mm decrease in difference T1-T2, compared to being female.						
**Overjet T1-T2, overbite T1-T2, UICD T1-T2, LIPD T1-T2, LIMD T1-T2, UIMD T1-T2, UAL T1-T2, LAL T1-T2 :**<br>Not associated to any prognostic factors.							
LICD; lower inter-canine distance, UICD; upper inter-canine distance, LIPD; lower inter-premolar distance, UIPD; upper inter-premolar distance, LIMD; lower inter-molar distance, UIMD; upper inter-molar distance, UAL; upper arch length, LAL; lower arch length.<br>Significance level: p-value<0.0042 (Bonferroni correction for 12 outcomes modelled).							

<table>
<tr><td colspan="8">Table S3. Prognostic factors for changes in PAR (T2-T1) and for LII grade at T2.</td></tr>
<tr><th></th><th>Prognostic factor</th><th>Coef.</th><th>Std. Err.</th><th>t</th><th>P>|t|</th><th colspan="2">[95% Conf. Interval]</th></tr>
<tr><td rowspan="4">PAR (T2-T1)</td><td>Lower fixed retainer at T2</td><td>-11.10</td><td>2.31</td><td>-4.80</td><td>0.000</td><td>-15.74</td><td>-6.56</td></tr>
<tr><td>Non-extraction treatment</td><td>5.97</td><td>1.63</td><td>3.66</td><td>0.001</td><td>2.70</td><td>9.24</td></tr>
<tr><td>Essix retainer at T1</td><td>5.38</td><td>1.36</td><td>-3.97</td><td>0.000</td><td>-8.11</td><td>-2.66</td></tr>
<tr><td colspan="7">- The presence of a lower fixed retainer at T2 correlates with 11.1 units decrease in difference in PAR score from T1 to T2, compared to no lower fixed retainer at T2.

- A non-extraction treatment correlates with 6.0 units increase in difference in PAR score from T1 to T2 compared to an extraction treatment.

- An upper Essix retainer at T1 correlates with 5.4 units decrease in difference in PAR score from T1 to T2 compared to an upper Begg retainer.</td></tr>
<tr><th></th><th>Prognostic factor</th><th>Odds ratio</th><th>Std. Err.</th><th>z</th><th>P>|t|</th><th colspan="2">[95% Conf. Interval]</th></tr>
<tr><td rowspan="3">LII grade at T2</td><td>LII at T1</td><td>0.08</td><td>0.06</td><td>-3.33</td><td>0.001</td><td>0.02</td><td>0.35</td></tr>
<tr><td>Lower fixed retainer at T2</td><td>732.95</td><td>1603.18</td><td>3.02</td><td>0.003</td><td>10.07</td><td>53321.82</td></tr>
<tr><td colspan="7">- A 1-mm increment in LII index at T1 correlates with a change of 0.08 in the odds of being in a better LII grade at T2.

- The presence of a lower fixed retainer at T2 correlates with a change of 733 in the odds of being in a better LII grade at T2.</td></tr>
<tr><td colspan="8">PAR; Peer Assessment Rating Index, LII; Little’s Irregularity Index.

Significance level: p-value<0.0033 (Bonferroni correction for 15 outcomes modelled).</td></tr>
</table>
